# Survey on the Awareness of the Use of Oropharyngeal Throat Packs in Dental Anesthesia: An International Online Survey

**DOI:** 10.7759/cureus.52320

**Published:** 2024-01-15

**Authors:** Atsuki Yamaguchi, Shota Tsukimoto, Hidetaka Kuroda, Uno Imaizumi, Norika Katagiri, Tomomi Katayama, Naotaka Kishimoto, Kanta Kido, Takahiro Abe, Takuro Sanuki

**Affiliations:** 1 Department of Dental Anesthesiology, Kanagawa Dental University, Yokosuka, JPN; 2 Department of Dental Anesthesiology, Dentistry and Graduate School of Medical and Dental Sciences, Niigata University, Niigata, JPN; 3 Department of Dental Anesthesiology, Hokkaido University, Sapporo, JPN; 4 Department of Oral and Maxillofacial Surgery, Kanagawa Dental University, Yokosuka, JPN

**Keywords:** general anesthesia, open-airway general anesthesia, dental treatment, oral surgery, oropharyngeal throat packs, pharyngeal throat packs, dental anesthesia

## Abstract

Introduction: Oropharyngeal throat packs (OPTPs) are frequently used to administer general anesthesia during oral surgery and dental procedures. However, the use of OPTPs has remained controversial, with concerns about their effectiveness, the potential for falling short of expectations, and the inherent risk of serious oversight in removing them. This study aimed to assess the awareness of dental anesthesiologists in the United States of America (USA) and Japan regarding the use of OPTPs.

Methods: An online questionnaire was distributed to 41 dental anesthesia education facilities in May 2023 and responses were obtained from 32 facilities.

Results: The responses to the questionnaire indicated that dental anesthesiologists in both the USA and Japan believe that using OPTPs during general anesthesia with airway securement is of significant importance, albeit with varying primary purposes for their application. In contrast, notable disparities were observed between the USA and Japan regarding the perceived importance and routine use of OPTPs during open-airway general anesthesia. In both countries, there is a common understanding that the residual risks of OPTPs are severe and that multiple preventive procedures are required.

Conclusions: The present study showed that dental anesthesiologists in the USA and Japan believed that the use of OPTPs was generally necessary for dental anesthesia. However, there was a difference in awareness between Japan and the USA regarding the importance of OPTPs for open-airway general anesthesia. Therefore, there should be a consensus among dental anesthesiologists in Japan and the USA on using OPTPs during open-airway general anesthesia in the near future.

## Introduction

Anesthesia management for oral surgery and dental procedures (dental anesthesia) has many unique features of perioperative airway management. This is because the surgical site is located in the upper airway. For example, oral intubation is commonly used for anesthesia in medical settings, whereas nasal intubation is frequently employed for dental anesthesia.

The insertion of oropharyngeal throat packs (OPTPs) is a clinical technique for airway management during dental anesthesia along with nasal intubation. OPTPs have been adopted for many years to achieve two purposes: 1) to stabilize the airway (maintain tidal volume by preventing air leaks), and 2) to prevent dental foreign bodies and/or water from entering the lower respiratory tract and esophagus [1-3]. In dental anesthesia, OPTPs are often inserted not only during general anesthesia with tracheal intubation but also under general anesthesia without tracheal intubation (open-airway general anesthesia: very similar to the intubated general anesthesia; however, there is no tracheal tube or supraglottic airway device placed in the airway, including deep sedation) [1-3].

In recent years, there has been growing momentum to review the use of OPTPs because of their potential effectiveness below expectations and the risk of inadvertent retention [4-6]. We have expressed our opinion that OPTPs remain important in clinical dental anesthesia despite this growing concern [2]. However, there is no evidence to support this personal opinion, as dental anesthesiologists’ views on the need for OPTPs in dental anesthesia practice have never been investigated. This study aimed to investigate dental anesthesiologists’ opinions in the United States of America (USA) and Japan regarding the importance and risks associated with using OPTPs in dental anesthesia practice. In addition, differences in awareness between the USA and Japanese dental anesthesiologists were examined.

## Materials and methods

Ethical guidelines

The online survey was approved by the Ethics Committee of Kanagawa Dental University (Approval No. 914; March 16, 2023). 

Structure and collection of questionnaires

The online survey was approved by the Ethics Committee of Kanagawa Dental University (Approval No. 914; March 16, 2023). A questionnaire consisting of 13 questions in three sections was developed using an online survey tool with Google Forms (Table 7 of Appendix). The questionnaire was originally created with reference to the questionnaire on the actual use of OPTPs in cleft centers developed by Curran et al. [6]. Section 1 involved obtaining consent to participate in the study. Sections 2 and 3 comprised six questions, each related to the awareness and practice of OPTPs under general anesthesia with tracheal intubation and open-airway general anesthesia (including deep sedation) within the field of dental anesthesia. Two versions of the same questionnaire were created: one in English and the other in Japanese. The questions were developed by four authors (AY, ST, HK, and TS) and reviewed for appropriateness by four authors (NK, KK, TA, and TS). All authors verified the functionality of the online survey tool and data collection accuracy. 

This study was conducted at all teaching facilities with residency programs affiliated with the American Society of Dentist Anesthesiologists (ASDA, n=9) and all teaching facilities of the Japanese Dental Society of Anesthesiology (JDSA, n=32). Contact information of the director of dental anesthesiology at each facility, listed on the websites of both societies, was collected. On May 1, 2023 (Japan time), the URL and QR codes for the online questionnaire entry screen were sent to ASDA facilities by e-mail and JDSA facilities by mail, accompanied by a request for cooperation in the study. We requested that the questionnaires be completed by only one participant from each facility. The response period was one month (until May 31, 2023). Owing to the lack of responses from the ASDA facilities, a reminder email was sent on June 15, 2023. Only facilities that answered all questions and fully completed the survey were included in this study.

Statistical analysis for comparison between the USA and Japan

Non-parametric data are presented as median (interquartile range) and were analyzed using the Mann-Whitney U-test. Observed frequencies were compared using Fisher's exact test, as appropriate. The SPSS (Statistical Package for Social Sciences, SPSS Inc, Chicago, Illinois, USA) was used to analyze the data. All significance tests were two-sided, and P-values of <0.05 were considered indicative of statistically significant differences. 

## Results

A total of 32 out of the 41 teaching facilities (six ASDA and 26 JDSA) participated in this study (response rate: overall, 78%; USA, 66.7%; Japan, 81.3%). All 32 teaching facilities fully completed the survey (survey completion rate: overall, 100%). 

Use of OPTPs in general anesthesia with airway securement by tracheal intubation

The results of the awareness of the use of OPTPs in general anesthesia with airway securement are presented in Table 1 . Both the USA and Japanese dental anesthesia teaching facilities believed that the use of OPTPs in general anesthesia with airway securement was very important (overall: 9.5 (8-9), USA: 10 (10-10), Japan: 8 (6.3-10)), and the frequency of remnant OPTPs was very low (overall: 0.5 (0-1), USA: 0 (0-0.8), Japan: 1 (0-1)). However, they considered the risk of remnant OPTPs extremely dangerous (overall: 10 (8-10), USA: 9 (3.5-10), Japan: 10 (8.3-10)). The number of preventive strategies required to avoid remaining OPTPs (such as "Included in surgical swab count," "Included on WHO sign-out checklist," and "End of throat pack kept long outside the oral cavity.") was overall 2 (2-3), USA: 2.5 (2-3), and Japan: 2 (2-3). No significant differences were observed between the USA and Japan in the importance of OPTPs (P=0.096), risk of remnant OPTPs (P=0.691), or the number of preventive strategies required to avoid OPTP remnants (P=0.691).

**Table 1:** Awareness of the use of OPTPs in general anesthesia with airway securement The difference in each questionnaire item between the two countries was evaluated by the Mann-Whitney U-test. USA, United States of America; OPTPs, oropharyngeal throat packs; IQR, interquartile range

Questionnaire item	Country	Median (IQR)	P-values (USA vs. Japan)
Importance of OPTPs	Overall	9.5 (8-9)	-
0: not important (not necessary); 10: very important (necessary)	USA	10 (10-10)	-
	Japan	8 (6.3-10)	0.096
Frequency of remnant OPTPs	Overall	0.5 (0-1)	-
0: never occurs; 10: very frequent (0 never occurs; 1-3: low frequency; 4-6: moderate frequency; 7-10: high frequency)	USA	0 (0-0.8)	-
	Japan	1 (0-1)	0.642
Risk of remnant OPTPs	Overall	10 (8-10)	-
0: very safe; 10: very dangerous (0: no risk; 1-3: low risk; 4-6: moderate risk; 7-10: high risk)	USA	9 (3.5-10)	-
	Japan	10 (8.3-10)	0.691
Number of preventive strategies required to avoid remnant OPTPs	Overall	2 (2-3)	-
	USA	2.5 (2-3)	-
	Japan	2 (2-3)	0.691

In most dental anesthesia teaching facilities in the USA and Japan, OPTPs were routinely inserted under general anesthesia with airway securement (overall: 29/32 (90.6%), USA: 6/6 (100%), Japan: 23/26 (88.5%) (Table 2). There was no statistically significant difference in the routine insertion of OPTPs between the USA and Japanese teaching facilities (P=1).

**Table 2:** Routine insertion of OPTPs in general anesthesia with airway securement The difference in the proportion of routine insertion between the two countries was evaluated by Fisher's exact test. USA, United States of America; OPTPs, oropharyngeal throat packs

Country	Yes, n (%)	No, n (%)	P-values (USA vs. Japan)
Overall (n=32)	29 (90.6)	3 (9.4)	-
USA (n=6)	6 (100)	0 (0)	-
Japan (n=26)	23 (88.5)	3 (11.5)	1

The primary reasons for inserting OPTPs under general anesthesia with the airway secured are presented in Table 3. Overall, the most common primary reason for inserting OPTPs under general anesthesia was to prevent aspiration and ingestion of water (19/32, 59.4%), followed by the prevention of aspiration and ingestion of solid foreign bodies (8/32, 25.0%). In Japanese teaching facilities, the most common primary reason was to prevent water aspiration and ingestion (17/26, 65.4%). However, in the USA, the aspiration and ingestion of solid foreign bodies were prevented (4/6, 66.7%, USA vs. Japan; P=0.023), and the reasons for this seem to differ depending on the country.

**Table 3:** Primary reason for the insertion of OPTPs in general anesthesia with airway securement The difference in the proportion of each reason between the two countries was evaluated by Fisher's exact test. USA, United States of America; OPTPs, oropharyngeal throat packs

Primary reason	Overall (n=32)	USA (n=6)	Japan (n=26)	P-values (USA vs. Japan)
Prevention of aspiration and ingestion of water (body or external fluids), n (%)	19 (59.4)	2 (33.3)	17 (65.4)	0.193
Prevention of aspiration and ingestion of solid foreign bodies (dental materials, instruments, etc.), n (%)	8 (25.0)	4 (66.7)	4 (15.4)	0.023
Gas leak prevention around tracheal tubes or supraglottic devices, n (%)	2 (6.3)	0 (0)	2 (7.7)	1
Prevention of displacement of tracheal tubes or supraglottic devices, n (%)	1 (3.1)	0 (0)	1 (3.8)	1
Not inserted, n (%)	2 (6.3)	0 (0)	2 (7.7)	1

Use of OPTPs in open-airway general anesthesia

The results of the awareness of the use of OPTPs in open-airway general anesthesia are presented in Table 4. Overall, using OPTPs was less important in open-airway general anesthesia (0 (0-5)). However, the USA teaching facilities believed that the use of OPTPs in open-airway general anesthesia was essential (9.5 (7.5-10)), and there was a statistically significant difference compared with Japanese teaching facilities (0 (0-2.8), P=0.011). Both the USA and Japanese teaching facilities considered the risk of remnant OPTPs in open-airway general anesthesia to be extremely dangerous (overall: 10 (8-10), USA: 8 (4.8-9.8), Japan: 10 (8-10)), although the frequency of remnant OPTPs was very low (overall: 0 (0-0.3), USA: 0.5 (0-1.8), Japan: 0 (0-0)). The number of preventive strategies required to avoid OPTP remnants in open-airway general anesthesia was 2 (2-3), USA 2.5 (2-3), and 2 (2-3). 

**Table 4:** Awareness of the use of OPTPs in open-airway general anesthesia The difference in each questionnaire item between the two countries was evaluated by the Mann-Whitney U-test. USA, United States of America; OPTPs, oropharyngeal throat packs; IQR, interquartile range

Questionnaire item	Country	Median (IQR)	P-values (USA vs. Japan)
Importance of OPTPs	Overall	0 (0-5)	-
0: not important (not necessary); 10: very important (necessary)	USA	9.5 (7.5-10)	-
	Japan	0 (0-2.8)	0.011
Frequency of remnant OPTPs	Overall	0 (0-0.3)	-
0: never occurs; 10: very frequent (0 never occurs; 1-3: low frequency; 4-6: moderate frequency; 7-10: high frequency)	USA	0.5 (0-1.8)	-
	Japan	0 (0-0)	0.278
Risk of remnant OPTPs	Overall	10 (8-10)	-
0: very safe; 10: very dangerous (0: no risk; 1-3: low risk; 4-6: moderate risk; 7-10: high risk)	USA	8 (4.8-9.8)	-
	Japan	10 (8–10)	0.723
Number of preventive strategies required to avoid remnant OPTPs	Overall	2 (2–3)	-
	USA	2.5 (2–3)	-
	Japan	2 (2–3)	0.392

Overall, the rate of routine insertion of OPTPs under open-airway general anesthesia was 18.7% (6/32, Table 5). However, the rates were 83.3% (5/6) in the USA and 3.8% (1/26) in Japan, and there were significant differences in the routine insertion of OPTPs under open-airway general anesthesia between the two countries (P<0.001, Table 5).

**Table 5:** Routine insertion of OPTPs in open-airway general anesthesia USA, United States of America; OPTPs, oropharyngeal throat packs The difference in proportion of routine insertion between the two countries was evaluated by Fisher's exact test.

Country	Yes, n (%)	No, n (%)	P-values (USA vs. Japan)
Overall (n=32)	6 (18.7)	26 (81.3)	-
USA (n=6)	5 (83.3)	1 (16.7)	-
Japan (n=26)	1 (3.8)	25 (96.2)	<0.001

The primary reasons for the insertion of OPTPs under open-airway general anesthesia are presented in Table 6. Overall, the most common primary reason was the prevention of aspiration and ingestion of solid foreign bodies (5/32, 15.6%), except “Not insert at all” (24/32, 75.0%). In the USA, only 16.7% (1/6) of teaching facilities did not insert OPTPs under open-airway general anesthesia; however, in Japan, the rate was 88.5% (23/26, P=0.002).

**Table 6:** Primary reason for the insertion of OPTPs in open-airway general anesthesia The difference in the proportion of each reason between the two countries was evaluated by Fisher's exact test. USA, United States of America; OPTPs, oropharyngeal throat packs

Primary reason	Overall (n=32)	USA (n=6)	Japan (n=26)	P-values (USA vs. Japan)
Prevention of aspiration and ingestion of solid foreign bodies (dental materials, instruments, etc.), n (%)	5 (15.6)	5 (83.3)	0 (0)	<0.001
Prevention of aspiration or ingestion of water (body or external fluids), n (%)	1 (3.1)	0 (0)	1 (3.8)	1
Other, n (%)	2 (6.3)	0 (0)	2 (7.7)	1
Not inserted, n (%)	24 (75.0)	1 (16.7)	23 (88.5)	0.002

## Discussion

The results of this study indicate that dental anesthesia teaching facilities in both the USA and Japan believe that using OPTPs during general anesthesia with airway securement is highly important, with differing primary purposes for their use. Compared between the USA and Japan, although there were significant differences in the awareness of the importance and routine use of OPTPs during open-airway general anesthesia, both dental anesthesia teaching facilities acknowledged the residual risk of OPTPs as highly perilous and deemed multiple precautions (such as "Included in surgical swab count," "Included on WHO sign-out checklist," and "End of throat pack kept long outside the oral cavity") necessary.

OPTPs have been commonly used during general anesthesia and head and neck surgeries, including dental and oral procedures, and have perceived benefits for patients, surgeons, and anesthesiologists [2,6,7]. These advantages include 1) preventing accidental ingestion and aspiration of dropped water and/or solid foreign bodies [1-3], 2) reducing postoperative nausea and vomiting (PONV) caused by increased gastric contents [2,3], and 3) stabilizing the airway during general anesthesia by preventing gas leaks and displacement of tracheal tubes or supraglottic devices (particularly in pediatric patients using cuffless endotracheal tubes or procedures requiring frequent head movement for surgical maneuvers) [2,3,8]. However, in recent years, several reports have advocated that OPTPs may not deliver all anticipated effects and could be harmful. The pros and cons of using OPTPs remain to be debated [2,4,5]. During general anesthesia, a comparative study between a group using OPTPs and a group abstaining from OPTPs reported a higher incidence and severity of sore throat in the group using OPTPs, although there are probably differences due to OPTPs insertion time and insertion method [2,9]. Compared with the group using OPTPs, another study has also reported that sore throat and dysphagia at 24 h postoperatively were significantly lower in the group abstaining from OPTPs, with no significant difference in PONV [2,10]. In addition, OPTPs are not only useless but can also cause adverse events. Over-insertion of OPTPs reportedly leads to acute tongue hypertrophy [11,12]. The most serious disadvantage of using OPTPs is the risk of accidental retention, potentially resulting in fatal airway obstruction after extubation when OPTPs are not removed [1,2,4,7,13]. A survey of all acute NHS trusts in the United Kingdom reported six cases of OPTP retention over three years [14]. However, the findings in this study showed that dental anesthesiologists in both the USA and Japan actively used OPTPs routinely, even though they were aware of the risk associated with forgetting to remove them. These results suggest that the advantages of using OPTPs surpass the risks and that implementing various precautionary procedures can mitigate the potential oversight of OPTP removal, although it is possible that the OPTP is being inserted according to conventional practice, without giving much thought to its advantages and disadvantages. Nevertheless, the use of OPTPs is undeniably more effective in physically preventing aspiration or accidental ingestion of foreign objects from the surgical site [3].

What is noteworthy about this study is that there was a difference in the awareness of the use of OPTPs between the USA and Japan during open-airway general anesthesia. The biggest advantage of using OPTP during open-airway general anesthesia is that it reduces water and/or dental foreign bodies from entering the lower respiratory tract and esophagus. On the other hand, the disadvantage is the risk of causing airway obstruction not only postoperatively, but also during the procedure. This study showed that dental anesthesiologists in the USA, but not in Japan, used OPTPs during open-airway general anesthesia. These results suggest that Japanese dental anesthesiologists believe that there are no benefits to using OPTPs during open-airway general anesthesia or that they see disadvantages in using OPTPs. In other words, although dental anesthesiologists in the USA maintain a consistent awareness of airway security, regardless of whether the patient is intubated or has an open-airway, there is insufficient evidence for the use of OPTPs in open-airway general anesthesia, including deep sedation, and there should be a consensus among dental anesthesiologist across the globe in using OPTPs during open-airway general anesthesia.

This study had several limitations. First, the survey did not investigate how dental anesthesiologists use OPTPs or how dental anesthesiologists prevent accidental OPTP retention. The disadvantages of using OPTPs include the possibility of causing postoperative sore throat, dysphagia, and acute tongue enlargement [2,9-12]. These complications are probably related to the material of the OPTPs and/or its insertion method (such as the use of sharp instruments and/or rough insertion methods), and whether OPTPs are an effective barrier depends on the packing method [2,7,15,16]. We believe that the incidence of these disadvantages can be reduced by using a soft material for the OPTPs, inserting it softly using appropriate instruments such as Magill forceps, and packing it carefully around the endotracheal tube [6]. Furthermore, we believe that the appropriate use of OPTPs will reduce the incidence of PONV due to blood intake. Second, it would have also been beneficial to track the specific preventive procedures performed by dental anesthesiologists in this survey. The NHS England suggests that it is a preventable event that should not occur if healthcare providers implement available precautions [17]. It has been reported that combining various preventive procedures (such as "Included in surgical swab count," "Included on WHO sign-out checklist," and "End of throat pack kept long outside the oral cavity") created throughout multiple positions (e.g., surgeons, anesthesiologists, and nurses) possibly reduces the incidence of forgetting to remove OPTPs to an extremely low level [18-20]. Filially, this survey has found a lack of consensus in using OPTP among dental anesthesiologists in Japan and the USA during open-airway general anesthesia. However, this survey did not clarify why there is such a difference.

## Conclusions

The present study showed that dental anesthesiologists in the USA and Japan believed that the use of OPTPs was generally necessary for dental anesthesia. However, there was a difference in awareness between Japan and the USA regarding the importance of OPTPs for open-airway general anesthesia. Therefore, there should be a consensus among dental anesthesiologists in Japan and the USA on using OPTPs during open-airway general anesthesia in the near future.

## Appendices

**Table 7:** Survey questionnaire

Introduction
Section 1. Informed consent form
1. Do you agree to cooperate in this study?
Agree/Disagree
Section 2. General anesthesia with airway securement by tracheal intubation or supraglottic devices
2. How important is oropharyngeal throat packs (OPTPs) insertion in general anesthesia with airway securement for dental anesthesia at your institution? 0: not important (not necessary); 10: very important (necessary)
0/1/2/3/4/5/6/7/8/9/10
3. How often does your institution consider the frequency of forgetting to remove OPTPs (remnant) in general anesthesia with airway securement for dental anesthesia? 0: never occurs; 10: very frequent (0 never occurs; 1-3: low frequency; 4-6: moderate frequency; 7-10: high frequency)
0/1/2/3/4/5/6/7/8/9/10
4. How does your institution consider the risk of forgetting to remove OPTPs (remnant) in general anesthesia with airway securement for dental anesthesia? 0: very safe; 10: very dangerous (0: no risk; 1-3: low risk; 4-6: moderate risk; 7-10: high risk)
0/1/2/3/4/5/6/7/8/9/10
5. How many preventive strategies do you believe are necessary to ensure that OPTPs are not inadvertently left behind in general anesthesia with airway securement?
1/2/3/4/5 or more
6. Does your facility currently routinely insert OPTPs in general anesthesia with airway securement for dental anesthesia?
Yes/No
7. What is the primary reason for routinely inserting OPTPs during general anesthesia with airway securement for dental anesthesia at your facility?
Prevention of aspiration and ingestion of water (body or external fluids)/Prevention of aspiration and ingestion of solid foreign bodies (dental materials, instruments, etc.)/Gas leak prevention around tracheal tubes or supraglottic devices/Prevention of displacement of tracheal tubes or supraglottic devices/Not inserted
Section 3. Open-airway general anesthesia including deep sedation
8. How important is OPTPs insertion in open-airway general anesthesia for dental anesthesia at your institution? 0: not important (not necessary); 10: very important (necessary)
0/1/2/3/4/5/6/7/8/9/10
9. How often does your institution consider the frequency of forgetting to remove OPTPs (remnant) in open-airway general anesthesia for dental anesthesia? 0: never occurs; 10: very frequent (0 never occurs; 1-3: low frequency; 4-6: moderate frequency; 7-10: high frequency)
0/1/2/3/4/5/6/7/8/9/10
10. How does your institution consider the risk of forgetting to remove OPTPs (remnant) in open-airway general anesthesia for dental anesthesia? 0: very safe; 10: very dangerous (0: no risk; 1-3: low risk; 4-6: moderate risk; 7-10: high risk)
0/1/2/3/4/5/6/7/8/9/10
11. How many preventive strategies do you believe are necessary to ensure that OPTPs are not inadvertently left behind in open-airway general anesthesia?
1/2/3/4/5 or more
12. Does your facility currently routinely insert OPTPs in open-airway general anesthesia for dental anesthesia?
Yes/No
13. What is the primary reason for routinely inserting OPTPs during open-airway general anesthesia for dental anesthesia at your facility?
Prevention of aspiration and ingestion of solid foreign bodies (dental materials, instruments, etc.)/Prevention of aspiration or ingestion of water (body or external fluids)/Other/Not inserted
